# High prevalence of previous dengue virus infection among first-generation Surinamese immigrants in the Netherlands

**DOI:** 10.1186/1471-2334-14-493

**Published:** 2014-09-10

**Authors:** Femke W Overbosch, Anneke van den Hoek, Janke Schinkel, Gerard JB Sonder

**Affiliations:** Department of Infectious Diseases, Public Health Service (GGD), Nieuwe Achtergracht 100, 1018 WT Amsterdam, The Netherlands; National Coordination Centre for Traveller’s Health Advice (LCR), Amsterdam, The Netherlands; Department of Internal Medicine, Division of Infectious Diseases, Tropical Medicine and AIDS, Academic Medical Center, Amsterdam, The Netherlands; Department of Medical Microbiology, Section of Clinical Virology, Academic Medical Center, Amsterdam, The Netherlands

**Keywords:** Dengue, Dengue virus infection, DENV, Seroprevalence, Prevalence, Suriname, Americas, Travellers, VFRs, Immigrants

## Abstract

**Background:**

A substantial portion of Dutch travellers is comprised of immigrants returning to their country of origin to visit friends and relatives (VFRs), including VFRs returning to dengue-endemic areas such as Suriname. Limited attention has been focused on dengue among immigrants, therefore it is unknown whether immigration has effect on the epidemiology of (severe) dengue among VFRs.

To get more insight in the seroprevalence of dengue among Surinamese immigrants, we conducted a seroprevalence study on a convenience sample of first-generation Surinamese immigrants living in the Netherlands.

**Methods:**

Blood samples were tested for IgG antibodies to DENV antigen serotypes (1, 2, 3 and 4). Gender, age, years lived in Suriname before immigration, history of yellow fever vaccination, and time between yellow fever vaccination and blood sample collection were examined as possible predictors for previous infection.

**Results:**

Of the studied 400 Surinamese travellers with a mean age of 52 years (range 18–89), 37% were male. Serology suggestive of past DENV infection was found in 325 individuals (81.3%; 95% CI: 77-85%). The time lived in Suriname before immigration was the only significant predictor for previous DENV infection.

**Conclusions:**

Most first-generation Surinamese immigrants have evidence of past DENV infection, probably comparable to Surinamese inhabitants. Whether this influences the number of cases of (severe) dengue when travelling requires more study.

## Background

Dengue is a mosquito-borne infection found in tropical and sub-tropical regions. The spectrum of clinical manifestations of dengue varies from a mild febrile self-limiting illness to a severe, potentially fatal disease. Substantial gaps remain in the basic understanding of the pathogenesis. Known is that there are four distinct, but closely related, serotypes of the virus that cause dengue (DENV-1, -2, -3 and -4). Recovery from infection by one serotype provides lifelong immunity against that particular type [1]. Hypothesized and strengthened by epidemiologic studies [2, 3] is that subsequent infection by other serotypes increases the risk of developing “severe dengue” also known as Dengue Haemorrhagic Fever.

In recent years, transmission in endemic areas has increased, predominantly in urban and semi-urban settings, and has become a major international public health concern. The disease is now endemic in more than 100 countries in Africa, the Americas, the Eastern Mediterranean, South East Asia and the Western Pacific, the latter two being the most seriously affected. Over 2.5 billion people (which is over 40% of the world’s population) are at risk [1]. The WHO estimates there may be 50–100 million dengue virus (DENV) infections worldwide every year. An estimated 500,000 people with severe dengue require hospitalisation each year, a large proportion of whom are children. About 2.5% of those affected die [1].

The Netherlands is not a dengue-endemic area; therefore Dutch citizens are not at risk of contracting a DENV infection in their home country. On the other hand, Dutch travellers are at substantial risk for DENV infection when travelling to endemic areas. A Dutch prospective study among short-term travellers conducted in 2006–2007 showed a serology-based attack rate of 1.2% and an incidence rate of 14.6 per 1000 person-months [4].

A substantial portion of Dutch travellers is comprised of immigrants returning to their country of origin to visit friends and relatives (VFRs), including VFRs returning to dengue-endemic areas such as Suriname, a former Dutch colony in the Caribbean (population 492,000 people) [5]. In 2010, 101,578 travellers from the Netherlands arrived in Suriname [6].

Although previous reports investigated the seroprevalence of dengue among people living in dengue endemic areas, limited attention has been focused on dengue among immigrants. Immigration to a non dengue endemic area causes deviation of exposure to DENV among immigrants compared to inhabitants of dengue endemic areas. Continuous exposure to DENV shifts to sporadic exposure during visits to the country of origin, which probably has consequences for the moment of encounter with a secondary, and potentially more severe, DENV serotype among immigrants. As far as we know, no research has been performed on dengue seroprevalence rates among Surinamese immigrants, nor among Surinamese nationals in their home country. Taking into account that different serotypes have been introduced in the Americas in past decades [7] and that predominant DENV serotypes can vary by year [8], immigration could influence the epidemiology of (severe) dengue among Surinamese immigrants. To get more insight in the seroprevelance among this group of travellers, we conducted a seroprevalence study among first-generation Surinamese immigrants living in the Netherlands who sought travel health advice at the Public Health Service’s Travel Clinic in Amsterdam.

## Methods

### Study population and design

A serum bank was used for this study, which consisted of blood samples of Surinamese first-generation immigrants who attended the Public Health Service’s Travel Clinic in Amsterdam from February 2008 to December 2011. These participants had been tested for immunity against hepatitis A or hepatitis B. Inclusion criteria for the hepatitis A and B immunity project were to have been born before 1970 or 1989, respectively. Data were collected concerning date of birth, gender, vaccination record, country of origin of both the participant and parents, and duration lived in country of origin. All participants had provided written informed consent to use the remains of the blood sample for anonymous scientific research on other infectious diseases. Following the rules stated in the Medical Research Involving Human Subjects Act (in Dutch: Wet Medisch-wetenschappelijk Onderzoek [WMO]), the informed consent letters for the hepatitis A and hepatitis B study were reviewed by the Medical Ethical Committee of the Academic Center of Amsterdam. The Medical Ethical Committee of the Academic Medical Center of Amsterdam approved all documents of the hepatitis A study (MEC 07/270), and reviewed all documents of the hepatitis B study and judged that evaluation of the hepatitis B study was not required (MEC 09/014). Therefore, we did not seek further waiver or approval for this specific dengue virus study.

### Laboratory

Blood samples were immediately stored at 6°C, then centrifuged and frozen at -80°C within 24 hours after collection. After being thawed they were tested for total immunoglobulin (IgG) antibodies to DENV antigen serotypes 1, 2, 3 and 4 by using an indirect ELISA (Panbio Diagnostics, Brisbane, Queensland, Australia) according to the manufacturer’s instructions. Test outcomes for DENV IgG antibodies were expressed as signal-to-cutoff ratios (s/co) and were interpreted according to the manufacturer’s instructions: ratios <0.9 were considered negative and thus as no evidence for past DENV infection, ratios ≥ 1.2 were considered reactive and thus as evidence for past DENV infection, and ratios 0.9-1.1 were considered boundary values.

### Statistical analysis

Test outcomes for DENV IgG antibodies which were considered boundary values according to manufacturer’s instructions, were allocated as negative for simplicity in statistical analysis.

SPSS for windows version 19.0 was used to obtain prevalence, prevalence ratios and 95% confidence intervals, by means of Poisson regression analysis with robust standard errors [9]. All variables with an overall p-value < 0.1 in univariate analysis were included in a multivariate analysis.

## Results

The serum bank included 400 unique samples of first-generation Surinamese immigrants living in the Netherlands, who intended to travel to (sub-)tropical countries. Eleven participants (2.8%) did not meet the age inclusion criteria for the original study; 3 participants from the hepatitis A project were born after 1970, and 8 participants from the hepatitis B project were born after 1989. We did not exclude these participants in our analysis. The mean age was 52 years (range 18–89) and 37% were male.

In 325 participants, DENV IgG was present, suggesting past dengue virus infection, making the serologic results suggestive of previous dengue virus infection in 325/400 (81.3%; 95% CI: 77-85%) participants. Six participants (1.5%) had DENV IgG levels at boundary values and therefore considered as negatives. Table 1 shows the prevalence (Ps) and prevalence ratios (PRs) with univariate and multivariate 95% confidence intervals. In univariate analysis, the prevalence in Surinamese > 60 years was significantly higher than in Surinamese aged 40 years or younger and positively related to the duration participants had lived in Suriname before immigration. In multivariate analysis, only duration of living in Suriname before immigration remained a significant predictor for previous DENV infection. The seroprevalence was not related to history of yellow fever vaccination or time between yellow fever vaccination and blood sample collection.

**Table 1:** **Characteristics of 400 first-generation Surinamese immigrants living in the Netherlands and their prevalence suggestive of previous dengue virus infection, February 2008 - December 2011**

		Total	DENV IgG		Univariable analysis*	Multivariable analysis
			Positive	P%	PR (95% CI)	PR (95% CI)
		n = 400	n = 325	81.3		
Sex						
	male	148	119	80.4	1	
	female	252	206	81.7	1.0 (0.92-1.1)	
Age (in years)						
	≤ 40	63	46	73.0	1	1
	41-50	112	90	80.4	1.1 (0.92-1.2)	1.5 (0.79-3.0)
	51-60	117	94	80.3	1.1 (0.92-1.3)	1.6 (0.84-3.2)
	≥ 61	108	95	88.0	**1.2 (1.0-1.4)**	1.4 (0.74-2.9)
Duration lived in Suriname (in years)						
	≤ 15	79	43	54.4	1	1
	16-20	112	88	78.6	**1.4 (1.2-1.8)**	1.4 (0.96-2.1)
	21-25	90	82	91.1	**1.7 (1.4-2.1)**	**1.6 (1.1-2.3)**
	≥ 26	115	108	93.9	**1.7 (1.4-2.1)**	**1.7 (1.1-2.5)**
History of yellow fever vaccination						
	no	258	211	81.8	1	
	yes (1 or 2)	142	114	80.3	0.98 (0.89-1.1)	
Time between (most recent) yellow fever vaccination and blood sample collection (in years)						
	≤ 5	41	29	70.7	1	1
	> 5-10	41	35	85.4	1.2 (0.96-1.5)	1.2 (0.94-1.4)
	> 10-15	41	36	87.8	1.2 (0.99-1.5)	1.2 (0.94-1.4)
	> 15	10	6	60.0	0.85 (0.49-1.5)	0.79 (0.47-1.3)

Participants attended the Public Health Service’s Travel Clinic in Amsterdam for pre-travel advice.

P = prevalence.

PR = prevalence ratio.

* Variables that were significant at p < 0.1 were selected for inclusion in the multivariable model.

## Discussion

In this study the prevalence of dengue virus IgG antibodies among first-generation Surinamese immigrants was 81%. Although seroprevalence studies among Surinamese inhabitants are not available, this is comparable with the results from seroprevalence studies among populations in Latin America [10–13]. It seems like immigration to a non dengue endemic country causes little difference in dengue seroprevalence between the Surinamese immigrants and the majority of populations living in dengue-endemic areas of the Americas and the Caribbean get infected with the dengue virus.

As expected, the seroprevalence of previous DENV infection in our study was positively related to the duration participants had lived in Suriname before immigration. This is in line with studies performed among persons living in dengue-endemic countries in Latin America where they found higher seroprevalence rates of DENV antibodies by increasing age, which can be seen as a marker for duration of exposure [13, 14].

A limitation of this study is that the used ELISA was not DENV serotype specific, making the number of participants at risk for a secondary infection with a different serotype still unidentified. If we however hypothetically assume that most of the DENV IgG positive participants had only one previous DENV serotype infection, a high number of Surinamese VFRs would be at risk for a secondary, potentially more severe dengue. Especially, as before 1963 only DENV-2 American genotype was reported in the Americas, but since then the region has been subject to repeated importation of new dengue serotypes and strains [7]. Over the last three decades a 4.6-fold increase in reported cases was observed in the Americas and even an 8.3-fold increase in DHF [15]. In the past ten years, all 4 dengue serotypes have been circulating in Suriname [16, 17].

The number of reported dengue cases or cases of severe dengue among Surinamese VFRs in Suriname or after return to the Netherlands, however, does not seem to be as high as one would expect. In 2010, the Pan American Health Organization (PAHO) reported only 113 clinical cases of dengue in Suriname (all lab confirmed) of which 20 cases were severe dengue including one death [16]. It is likely, though, that a substantial number of under- or misdiagnosis occurs in Suriname as only lab-confirmed dengue virus infections have been reported. Also, the number of dengue cases or severe dengue infections in the Netherlands is not clear as dengue is not a notifiable disease in this country. However, severe dengue is extremely rare among Surinamese travelers returning to the Netherlands who attend the Academic Medical Center in Amsterdam, which serves an important portion of Surinamese immigrants in the Netherlands (in 2008, 338,000 Surinamese lived in the Netherlands of whom 20% lived in Amsterdam) [18]. This low incidence of severe dengue among Surinamese could possibly be explained by host factors. Guzman’s review summarises host factors that may reduce the risk of severe disease during a second dengue virus infection, which include race, second- or third-degree malnutrition, and polymorphisms in the Fcγ receptor and vitamin D receptor genes [19]. Perhaps one or more of these factors can be applied to the Surinamese population.

Our study has some other limitations. Cross-reactivity between the dengue virus and other flaviviruses cannot be excluded. However, in our study, cross-reactivity with yellow fever vaccination is not likely since no relation was found with either previous yellow fever vaccinations or time between yellow fever vaccination and sample collection. This is in agreement with the results of a study on the incidence of dengue virus infection among Dutch short-term travellers [4].

Second, the data used for this study is extracted from a serum bank of travellers visiting the Public Health Service’s Travel Clinic in Amsterdam. The serum bank population may not be representative for the population of first-generation Surinamese living in the Netherlands and travelling to Suriname.

Third, data about duration lived in Suriname was self-reported by the participants. In part, this was also the case for history of yellow fever vaccination. Available data concerning these variables could thus deviate from the actual data, however, we do not consider these limitations to have significantly affected our findings.

Lastly, frequency and duration of stays in endemic areas after immigration was not incorporated as variables as these data was missing in the serum bank. This could have been of influence to the seroprevalence we found.

## Conclusions

Most first-generation Surinamese immigrants living in the Netherlands display evidence of past dengue virus infection, probably comparable to Surinamese inhabitants. Surinamese immigrants have possibly been infected with fewer DENV serotypes, but whether this influences the number of cases of (severe) dengue is unknown. Incidence rates of severe dengue among Surinamese, a seroprevalence study among Surinamese inhabitants and serological tests which can discern the different DENV serotypes should be performed to uncover immigrants as a potential risk group for (severe) dengue. This could be of great importance for the development of specific dengue preventive policies.

## Abbreviations

DENV: Dengue Virus
WHO: World Health Organization
VFRs: Visiting Friends and Relatives
MEC: Medical Ethical Committee
ELISA: Enzyme-linked Immunosorbent Assay
IgG: Immunoglobulin G
CI: Confidence Interval
P: Prevalence
PR: Prevalence Ratio
PAHO: Pan American Health Organization.
